# Determinants for adherence to continuous positive airway pressure therapy in obstructive sleep apnea

**DOI:** 10.1371/journal.pone.0189614

**Published:** 2017-12-18

**Authors:** Anne Roed Jacobsen, Freja Eriksen, Rasmus Würgler Hansen, Mogens Erlandsen, Line Thorup, Mette Bjerre Damgård, Martin Glümer Kirkegaard, Klavs Würgler Hansen

**Affiliations:** 1 Diagnostic Centre, Regional Hospital Silkeborg, Silkeborg, Denmark; 2 Section for Biostatistics, Department of Public Health, Aarhus University, Aarhus, Denmark; 3 Interdisciplinary Research Unit, Elective Surgery Centre, Regional Hospital Silkeborg, Silkeborg, Denmark; 4 Sleep Disorders Clinic, Elective Surgery Centre, Regional Hospital Silkeborg, Silkeborg, Denmark; Technische Universitat Munchen, GERMANY

## Abstract

**Background:**

Continuous positive airway pressure (CPAP) therapy is an efficacious treatment for patients diagnosed with obstructive sleep apnea (OSA). However, there are only few data on long-term adherence. The aim of this study is to quantify the extent of non-adherence and describe the clinical characteristics.

**Methods:**

A retrospective study including 695 patients with newly diagnosed OSA and prescribed CPAP therapy within an inclusion period of 14 months. All patients were offered free of charge individually adjusted CPAP therapy. Data on comorbidity, medication, BMI and Epworth Sleepiness Score (ESS) were obtained by questionnaires and consultation with an otorhinolaryngeal specialist.

**Results:**

The median follow-up time after initiating CPAP therapy was 3.0 (range 2.4–3.6) years. An adherence rate of 89% was found for severe OSA, 71% for moderate OSA and 55% for mild OSA. 18% initiated humidification. Patients adherent to CPAP had a significantly higher Body Mass Index (BMI), Apnea Hypopnea Index (AHI), Oxygen Desaturation Index (ODI) and ESS compared to non-adherent patients. Furthermore, adherence was associated with a higher frequency of observed interrupted breathing, a less frequent use of hypnotic drugs, fewer smokers, and they were more often offered humidification. Age, gender and comorbidity were not significantly associated with adherence. In a Cox model only AHI (Hazard Ratio (HR) 0.963, p < 0.001), ESS (HR 0.939, p = 0.001) and smoking (HR 1.576, p = 0.022) were independently associated with CPAP non-adherence.

**Conclusions:**

The severity of OSA, subjective daytime sleepiness and smoking status are independently related to adherence to CPAP therapy.

## Introduction

Obstructive sleep apnea (OSA) is a common condition associated with impaired cognitive function, decreased quality of life due to daytime sleepiness and increased risk of traffic accidents and cardiovascular events [1–2]. If used consistently, continuous positive airway pressure (CPAP) therapy is an efficacious treatment. Alternative treatment can only infrequently eliminate the need for CPAP, primarily in case of a larger weight loss, application of mandibular advancement devices, positional therapy and upper airway stimulation. The latter treatment modality is not introduced in clinical practice in Denmark. However, the effectiveness of the treatment is limited by variable adherence to prescribed CPAP therapy [3–5]. Non-adherence can be reduced by individual adjustments and titration of the CPAP therapy, including the type of mask and humidification [4]. Only few studies report data on long-term CPAP adherence and no single factor has been consistently identified as predictive of adherence [5–6]. The aim of this study is to quantify the extent of non-adherence within the current possibilities of individual adjusted treatment and describe the clinical characteristics associated with non-adherence.

## Methods

The study was approved by the Danish Health Authority and Danish Data Protection Agency in Central Region Denmark (Case number 1-16-02-30-13) with permission to store non-anonymized data in a secured research database. Data must be destroyed or anonymized before 01-05-2023.

This retrospective observational study included patients referred to the Outpatient Clinic for Sleep Disorders in Silkeborg Hospital. The clinic offers free of charge ambulatory diagnostic Cardio Respiratory Monitoring (CRM) and subsequent treatment to patients referred both from primary physicians and hospital departments. All the included patients underwent a diagnostic CRM at home. 70% of the patients were measured with the Philips Stardust II Sleep Recorder and data were analyzed by the Alice PDX software. The remaining 30% were measured with approved equipment from the referring physician, but the exact type is unknown to us.

The patients filled in a questionnaire regarding sleep related symptoms, health history, medication, smoking, height, weight and Epworth Sleepiness Score (ESS) for quantifying daytime sleepiness. In addition, an otorhinolaryngeal specialist supplemented with problem orientated questions focusing on comorbidity, snoring and observed interrupted respiration at night. Finally, a clinical examination was performed, before the patients were offered CPAP therapy. The database included 695 patients with newly diagnosed OSA from the 1st of January 2012 to the 28th of February 2013. During this period the clinic consistently used the Philips SystemOne REMstar autoCPAP and masks from Philips with a selection of 3 types of nasal masks, 3 full face masks and 1 with a nostril tube. All patients were seen by a sleep disorder nurse specialist one month after initiation of the treatment, followed by individual intervals with adjustments of the combination of CPAP, type of mask and supplementary humidification if considered necessary, until treatment was well-tolerated by the patient. Eventually patients were reassessed in the clinic at least once a year. Patients, who needed a replacement during the period of follow-up, were offered a ResMed S9 Autoset CPAP device due to change of supplier. The medical files of all patients were reviewed. A patient was considered non-adherent if the patient decided to stop treatment due to intolerance or subjective lack of benefit or if the patient was lost to follow-up, i.e. did not respond to any communication or requested new masks, tubes or filters. The usage among the adherent patients was evaluated from the Secure Digital (SD) card of the CPAP device at last available annual follow-up.

An apnea is defined as a pause in breathing lasting at least 10 seconds, while hypopnea is defined as hypoventilation combined with oxygen desaturation > 4%. The Apnea Hypopnea Index (AHI) is the average number of apneas and hypopneas per hour. The severity of OSA is commonly classified as mild (5 ≤ AHI < 15), moderate (15 ≤ AHI < 30) or severe (AHI ≥ 30). Oxygen Desaturation Index (ODI) is the average hourly number of episodes with desaturation > 4%.

### Statistical analysis

Variables are presented as mean ± SD except for AHI, ODI and fraction of nights with CPAP (median and range or 25–75 interquartile range), which were not normally distributed as evaluated by Q-Q plots. Unpaired t-test or Mann Whitney test as appropriate was used for comparison between groups. Correlation between the usage time and strata of AHI was tested by calculation of Spearman´s rho. Categorical data were analysed by chi-square test. Adherence to CPAP therapy and introduction of humidification was evaluated by Kaplan-Meier analysis. The difference in Kaplan-Meier CPAP therapy survival curves for strata of AHI was tested with a log rank test. A Cox proportional hazard model with CPAP non-adherence as dependent variable was used to evaluate the independent contribution of gender, BMI, use of humidification, AHI, ODI, ESS and use of hypnotic drugs. Humidification was entered in the Cox model as a time dependent variable. The statistical program SPSS ver. 20.0 was used.

## Results

**Fig 1:**
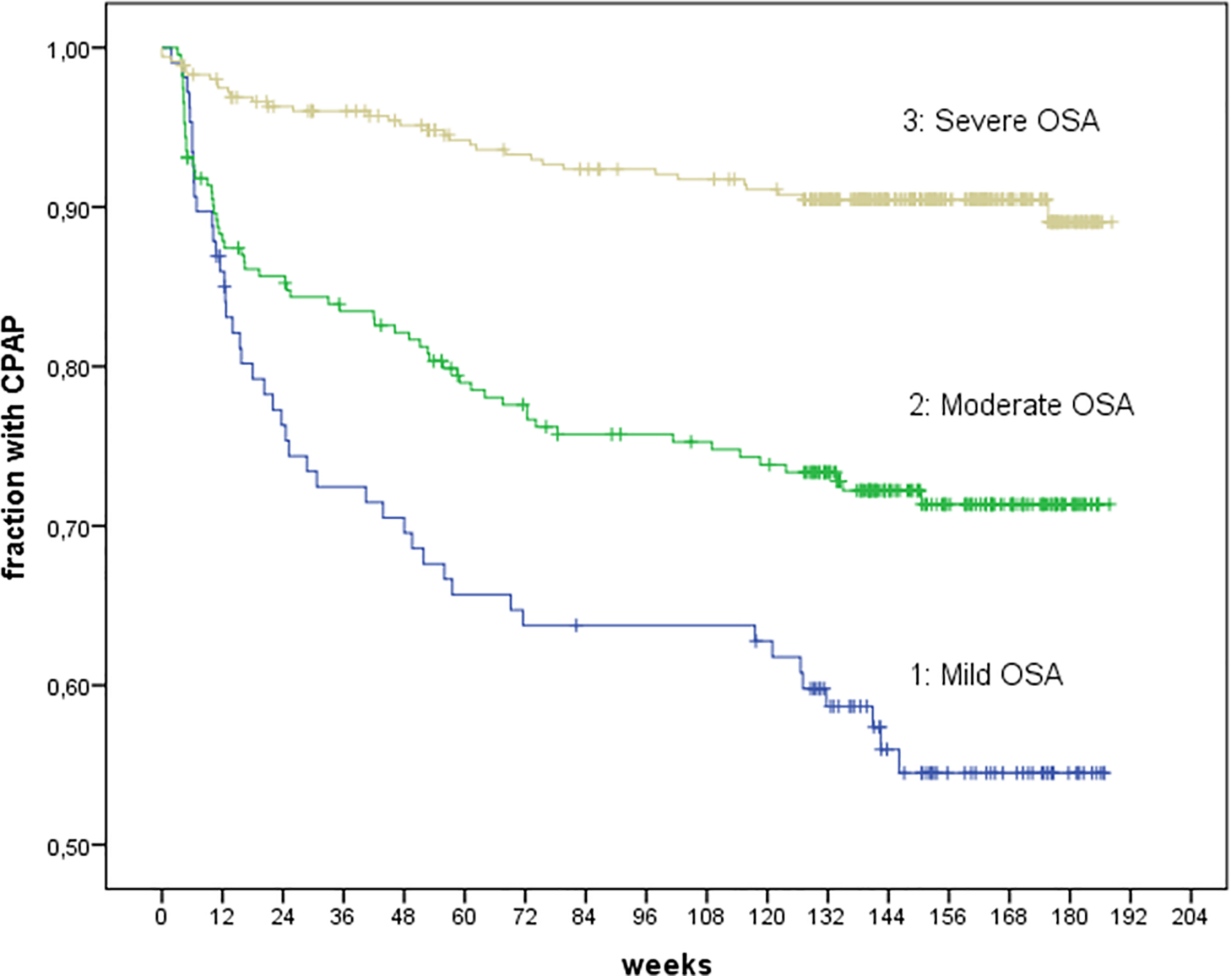
Kaplan-Meier plot. Kaplan-Meier plot showing the fraction of patients adherent to CPAP therapy for three strata of AHI; mild, moderate and severe OSA.

The period of observations concerning CPAP status started from the initiation of the CPAP therapy until august 1st 2015 resulting in a median of 1105 days (range 890–1318) corresponding to median 3.0 years (range 2.4–3.6). 29 patients ended the CPAP therapy due to medical reasons. The main cause was a large weight loss among 16 patients, 11 of them after gastric bypass surgery. Furthermore, 6 patients were effectively treated with a mandibular advancement device, 4 with tonsillectomy or nasal septum surgery, 2 after positional therapy and 1 for other reasons. Among the remaining 666 patients, 142 were non-adherent despite continued indication for treatment. 113 of these patients informed the clinic that they had stopped the treatment due to intolerance to CPAP therapy or no subjective benefit. Finally, 29 poorly compliant patients were eventually lost to follow-up. The follow-up period with CPAP therapy for the cohort was censored if the treatment was no longer indicated (n = 29), if patients were referred to Respiratory Center West, Aarhus University Hospital, because of possible central sleep apnea and/or Cheyne-Stokes respiration (n = 25), or to a neurological department (n = 2), in case of death (n = 6) or emigration (n = 1) and if the patients moved to another clinic outside the region (n = 3). In 21 of the 29 patients, who were no longer considered candidates for CPAP therapy, this was documented by a final CRM. For these patients median baseline AHI was 28 (range 11–116) and final AHI was 6 (range 2–13) (p < 0.001). The overall adherence rate to CPAP was 77.7%. The Kaplan-Meier curve (n = 106) showed 54.5% adherence for mild OSA (n = 106), 71.3% adherence for moderate OSA (n = 234) and 89.1% for severe OSA (n = 355)(p < 0.001) (Fig 1).

**Fig 2:**
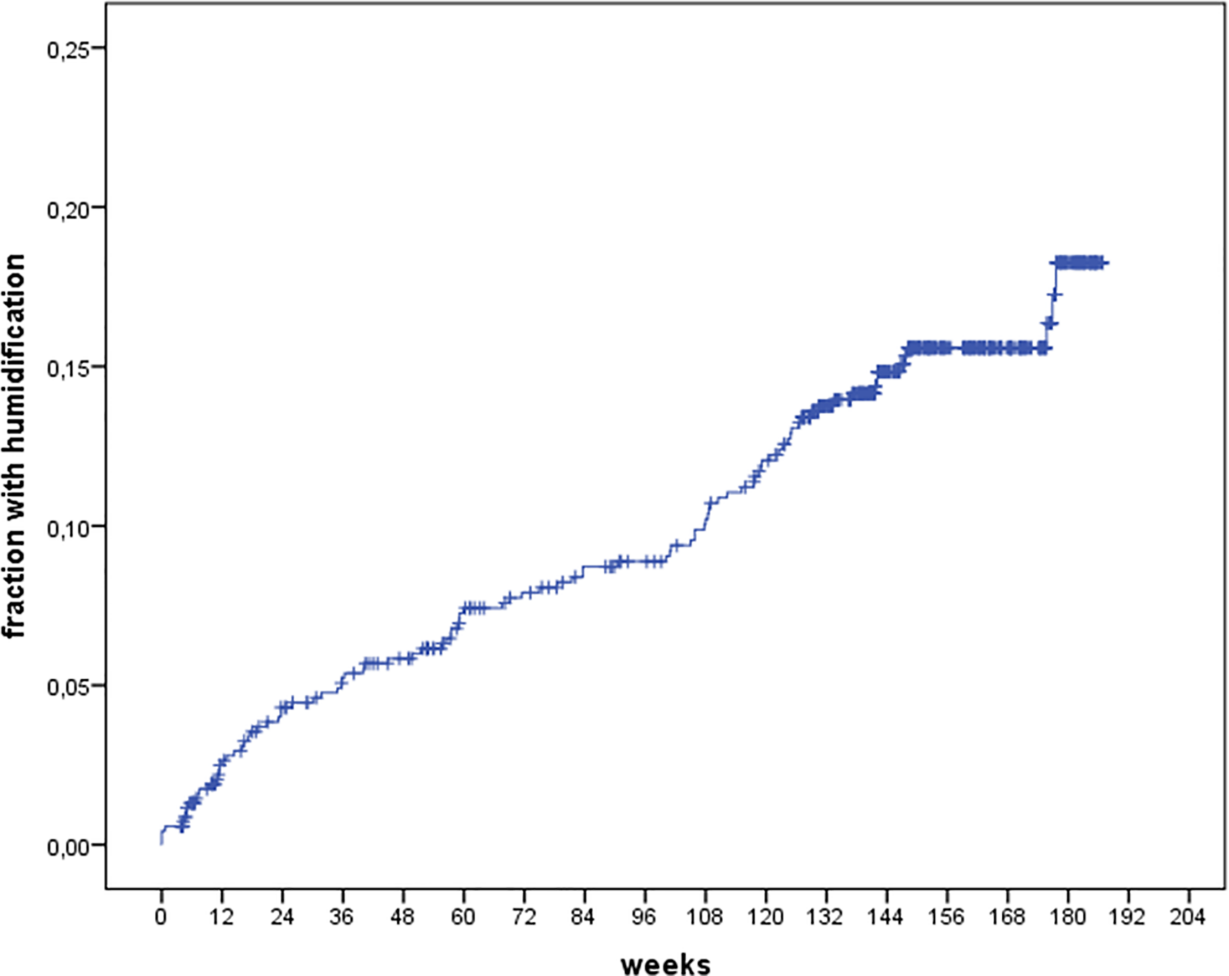
Kaplan-Meier plot (1—survival) of the fraction of patients with CPAP treatment and humidification.

Humidification was offered to 18.3% of the patients according to the Kaplan-Meier analysis. The time for introduction of humidification was distributed evenly during the observation period (Fig 2).

At baseline patients adherent to CPAP therapy had a significant higher Body Mass Index (BMI), AHI, ODI and ESS compared to non-adherent patients and were more frequently offered humidification (Table 1). Furthermore, the adherent group also reported less frequent use of hypnotic drugs, fewer smokers and a higher fraction of patients with observed interrupted breathing and snoring. Age, gender and comorbidity were not significantly associated with consistent use of CPAP therapy.

**Table 1:** Baseline clinical characteristics of patients diagnosed with OSA.

	Persistent CPAP (n = 553)	Non-adherence (n = 142)	P-value
Male (%)	75% (412/553)	75% (106/142)	NS
Age (years)	53.5 ± 11.9	51.8 ± 13.4	NS (p = 0.15)
Body weight (kg)	98.1 ± 22.9 (n = 525)	92.0 ± 20.0 (n = 135)	P = 0.004
BMI (kg/m^2^)	31.4 ± 6.6 (n = 523)	29.3 ± 5.6 (n = 133)	P = 0.001
Humidification	16% (88/553)	7% (10/142)	P = 0.007
AHI (events/h)	34 (5–116)	21 (6–113)	P<0.001
ODI (events/h)	24 (0–118) (n = 548)	13 (0–144) (n = 140)	P<0.001
ESS total score	11.0 ± 5.0 (n = 535)	8.8 ± 4.6 (n = 134)	P<0.001
Snoring	84% (465/551)	77% (110/142)	P = 0.05
Observed interrupted breathing	70% (386/548)	58% (82/142)	P = 0.004
Nycturia	75% (405/538)	75% (101/137)	NS (P = 0.78)
Antihypertensive treatment	40% (220/553)	39% (55/142)	NS (P = 0.82)
Hypnotic drugs	7% (37/550)	13% (18/139)	P = 0.016
Smoking	23% (125/544)	33% (46/139)	P = 0.014
Diabetes	10% (53/553)	5% (7/142)	NS (P = 0.08)
Pulmonary disease	8% (44/553)	12% (16/142)	NS (P = 0.21)
Cardiovascular disease	7% (35/553)	10% (14/142)	NS (P = 0.14)
Cerebrovascular disease	4% (20/553)	5% (7/142)	NS (P = 0.47)

AHI: Apnea Hypopnea Index, BMI: Body Mass Index, ESS: Epworth Sleepiness Score, ODI: Oxygen Desaturation Index. Data are shown as percent, numbers, mean +/- SD or median (range). P-values are not corrected for multiple testing.

All data regarding the independent variables in the Cox proportional hazard model were available for 620 patients (123 non-adherent). The Cox analysis showed that AHI, ESS and smoking status were independently related to CPAP-adherence (Table 2). Higher AHI and ESS improved and smoking decreased adherence.

**Table 2:** Cox proportional hazard model for non-adherence to CPAP therapy.

Independent Variable	P-value	Hazard ratio (95% Cl)
Gender (male = 1)	0.13	0.716 (0.465–1.103)
BMI	0.75	0.994 (0.960–1.030)
AHI	< 0.001	0.963 (0.943–0.982)
ESS	0.001	0.939 (0.904–0.974)
ODI	0.42	0.992 (0.974–1.011)
Hypnotic drugs at baseline (drug use = 1)	0.25	0.725 (0.421–1.248)
Humidification (time dependent exposure) (humidification = 1)	0.41	1.368 (0.653–2.870)
Observed interrupted breathing (yes = 1)	0.058	1.498 (0.986–2.274)
Snoring (yes = 1)	0.95	1.017 (0.613–1.686)
Smoking (yes = 1)	0.022	1.576 (1.066–2.330)

AHI: Apnea Hypopnea Index, BMI: Body Mass Index, ESS: Epworth Sleepiness Score, ODI: Oxygen Desaturation Index.

Data regarding AHI during treatment and usage time was based on all adherent CPAP users excluding patients who stopped treatment due to effective alternative intervention (n = 29), were referred to a university clinic for bi-level CPAP or to a neurological department (n = 27) or were transferred to a clinic outside the region (n = 3). The median achieved AHI with CPAP therapy was 2.5 (IQR 1.5–3.8). The CPAP device was used for a median of 96.4% (IQR 85.5–99.5) of all nights (mean 6.1 ± 1.5 hours per night). Calculated as the mean of all nights CPAP usage was 5.5 ± 1.9 hours. These data presented separately for all three strata of AHI are shown in Table 3. Follow-up AHI, fraction of night with CPAP and average use of CPAP per night all correlated significantly with strata of AHI at baseline.

**Table 3:** AHI at baseline and follow-up and CPAP usage time for persistent CPAP users.

	AHI baseline	AHI follow-up	Fraction of night with CPAP (%)	Average use during night with CPAP (hours)	Average use of CPAP for all nights (hours)	Fraction (%) of patients who use CPAP > 70% of the nights and > 4 hours per night
**Mild OSA (n = 56)**	12.9 (11.1–13.8)	2.1 (1.2–3.1) (n = 54)	93.3 (78.0–98.4) (n = 54)	5.7 ± 1.6 (n = 54)	5.0 ± 2.0 (n = 54)	75.9% (41/54)
**Moderate OSA (n = 146)**	22.0 (18.8–24.9)	2.3 (1.4–3.3) (n = 144)	96.1 (83.5–99.3) (n = 144)	6.1 ± 1.2 (n = 144)	5.4 ± 1.7 (n = 144)	82.6% (119/144)
**Severe OSA (n = 292)**	47.6 (37.5–62.6)	2.9 (1.7–4.3) (n = 288)	97.4 (87.7–99.7) (n = 288)	6.2 ± 1.5 (n = 288)	5.7 ± 1.9 (n = 288)	84.4% (243/288)
**Spearman´s rho**	Not relevant	0.18 P<0.001	0.15 P = 0.001	0.10 P = 0.036	0.12 P = 0.008	0.06 P = 0.2

AHI: Apnea Hypopnea Index. Data are shown as median (25–75 interquartile range), mean +/- SD or percent and numbers.

## Discussion

We found an overall adherence rate of 78% after a median follow-up time of 3 years within the current options of individually adjusted CPAP therapy. A direct comparison to previous studies is rather complex due to different follow-up time and composition of patients. The study by Kohler et al [6] included 639 patients with sleep-disordered breathing and median follow-up time was 3.9 (1.5–6.9) years. The percentage of patients adherent to CPAP after 5 and 10 years was 81% and 70%, respectively. Patients with missing medical records and those lost to follow-up (n = 197), corresponding to 24% of the population, were excluded from the analysis in the study by Kohler. It is plausible that the fraction of non-compliant patients is higher in this group and the adherence rate may have been overestimated. Galetke et al [7] followed 303 patients for a median follow-up time of 13 months and 63% were still using the CPAP device regularly. An older study by Krieger et al [8] followed 608 patients (575 with AHI > 15) for an average of 3.2 years. The adherence rate for patients with AHI > 15 was slightly above 90% and approximately 65% for patients with AHI ≤ 15. The largest study of CPAP adherence is reported by McArdle et al [9] who followed 1,110 patients commencing CPAP between 1986 and 1997. The follow-up period was relatively short (median 1.8 years). Both AHI, ESS and a snoring history were independent predictors of adherence. Adherence for patients with both AHI > 30 and ESS > 10 was slightly above 70%. The average CPAP running time for all nights varies between 5.6 and 6.2 hours [6,8,9]. A correction factor of 0.95 has been suggested to calculate the time with effective mask pressure as presented in our study [8]. Thus the mean usage of 5.5 hours (calculated from all nights) in the study corresponds very closely to previous results. Overall, the findings of present studies advocate that it is difficult to further increase the adherence rate or usage time described in current data [6–20].

The study showed that both AHI and daytime sleepiness were independently associated with CPAP adherence. This finding is in contrast to the study by Kohler et al, where no influence of daytime sleepiness reported as ESS was found [6]. AHI and ODI are closely related parameters describing the same aspects of the severity of the disease. The significant association between adherence and a higher AHI confirms the conclusions in the study by Kohler et al [6], where the severity of sleep-disordered breathing (evaluated by ODI only) was found to determine long-term adherence. The results suggest a natural selection causing patients with the most severe OSA to have a superior adherence to CPAP therapy. It makes sense to indicate a subjective effect of CPAP therapy, which cannot be assessed by a simple statement regarding daytime sleepiness. The hazard ratio of 0.96 per unit increase in AHI in our study corresponds to the hazard ratio of 0.97 per unit increase in ODI in the study by Kohler et al [6]. This implicates that a patient with AHI of 30 compared to a patient with AHI of 15 is expected to have a 57% (= 0.963^15^, 95% CI 41–76%) lower risk of quitting CPAP therapy, i.e. a risk reduction of 43%. The clear separation of adherence rate to strata of AHI indicates that comparison between studies should be within each stratum to overcome the influence of different composition of patients. AHI and ODI are related to BMI, but BMI was not independently associated with CPAP adherence in our study. The study by Kohler et al [6] does not have any data on BMI, but finds no association regarding neck circumference. We find only a small percentage of 4.2% (29 out of 695) of patients diagnosed with OSA, who discontinued CPAP therapy because of another effective treatment, which in most cases consists of a larger weight loss after gastric bypass surgery.

We did not reclaim the CPAP device in cases of suboptimal usage, if the patients wanted to continue because of some subjective benefit. The average usage per night in our population is comparable to other studies. In addition, we present the more appropriate information about the percentage of night, when CPAP is used and the average hour used in these nights. Interestingly both the adherence rate and the percentage of nights with CPAP depends on strata of AHI. Patients with milder degrees of OSA are not only more likely to stop the treatment, but those who continue demonstrate lower usage period. Only 84% of persistent CPAP users with severe AHI at baseline manage to use the device both > 70% of the nights and > 4 hour per night as commonly considered the lower limit of clinical meaningful usage [9]. This finding highlights the need for cardiovascular outcome studies to report the results from both the intention to treat with CPAP and per protocol analysis.

We found that a smoker had a 58% higher risk of terminating CPAP therapy. The estimate is imprecise since the confidence limit is very wide (7 to 223%). Smokers may be more susceptible to upper airway discomfort of CPAP therapy and/or less likely to follow advice from health care providers. An observation in favour of the latter theory comes from McArdle et al, who report that smokers with OSA were more likely than non-smokers to refuse CPAP therapy even before they have tried it [9].

The frequency of patients offered humidification was higher in persistent CPAP users which can be explained by a “survivor” bias. Patients were considered candidates for humidification evenly during follow-up and for this reason inevitable have been persistent CPAP users for some time. When properly evaluated with time dependent Cox analysis this treatment did not influence on adherence to the therapy. The effect of humidification should be evaluated by randomized studies. Although humidification alleviates upper airway symptoms, no consistent effect on adherence to CPAP therapy has been proven [21–22].

The striking decline in CPAP adherence during the first few months in particular for patients with mild or moderate OSA illustrate a window of opportunity [9]. A considerable fraction of patients decline CPAP use very quickly, even before having tried most common interventions to optimize compliance. It is unknown whether humidification from the start of CPAP therapy, which is now the standard in our clinic, can reduce early dropout.

Only one randomized, long-term study regarding the effect of OSA on cardiovascular events has been performed. The recently published Sleep Apnea Cardiovascular Endpoint (SAVE) study reported no effect of CPAP therapy as a secondary prophylactic intervention for prevention of a composite of cardiovascular events [23]. As a secondary endpoint analysis the subgroup of patients adherent to CPAP therapy ≥4 hours per night had a reduced risk of stroke. Most previous studies compare patients adherent to CPAP therapy to non-adherent patients, where several find an increased frequency of cardiovascular mortality among non-adherent patients [24–27]. This could be confounded if a lifestyle leading to an increased probability for non-adherence also is associated with a higher comorbidity and unhealthy lifestyle in general, such as smoking. We did not find that non-adherent patients had a more conspicuous comorbidity at baseline than the group of adherent patients which is in accordance with studies comparing cardiovascular events in patients compliant and non-compliant to CPAP therapy [24,25,27].

A strength of this study is that all the patients have individual adjustment of the CPAP therapy. Furthermore, there is a considerable follow-up time and detailed baseline data about comorbidity available. We have identified the group of patients, who quit the treatment due to another relevant treatment and have included patients lost to follow-up as non-adherent patients, thereby giving a realistic image of the true non-adherent patients.

The study is retrospective and therefore limited by the impossibility of personally interviewing the patients, who discontinued the treatment due to personal causes. Hence, some aspects could not be described, e.g. alcohol use, psychiatric disease, social position, relationship status (single or non-single) and possible perception of being stigmatized or discomfort from CPAP therapy. These suggestions could not be proved as reasons for non-adherence.

In conclusion, our study includes a large cohort of patients with newly diagnosed OSA. 88% are adherent to the prescribed CPAP therapy after a median follow-up time of 3 years. The severity of OSA evaluated by AHI, subjective daytime sleepiness and non-smoking is independently related to adherence to CPAP therapy.
